# Supplementary material for: Asynchronous magnetic resonance elastography: Shear wave speed reconstruction using noise correlation of incoherent waves
Source: Magn Reson Med. 2022 Oct 27;89(3):990–1001. doi: 10.1002/mrm.29502 (PMC9792433; doi:10.1002/mrm.29502)
Supplement: Supplementary file 1 — DATA S1 MATLAB code used to generate the numerical simulations of Figure 1 [file MRM-89-990-s002.zip › k-Wave/helpfiles/benchmark.html]

benchmark :: Functions (k-Wave)


# benchmark

Run performance benchmark.

## Syntax

```
benchmark
```

## Description

`benchmark` performs an analysis of the time taken to run simulations using `kspaceFirstOrder3D` with increasing grid sizes. The default simulation uses a heterogeneous absorbing medium, a binary sensor mask with 100 points, 1000 time steps, `'PlotSim'` set to false, and grid sizes varying from 32^3 to 256^3. The times are computed from 3 averages and the function stops when memory errors are encountered or the benchmarking is complete. The computational times and settings are stored in a MATLAB formatted binary file using `save`.

The computation times and relative speed running `benchmark` using an 8-Core CPU compared to using an NVIDIA Tesla C2070 GPU (graphics processing unit) are shown below. For the GPU computations, the maximum possible grid size is limited by the memory available on the GPU. Note, before running, the MATLAB workspace is cleared and any open MATLAB windows are closed.
